# Cross-species transmission alert: a novel canine-raccoon dog coronavirus infecting an Amur Tiger in China

**DOI:** 10.3389/fmicb.2026.1764349

**Published:** 2026-02-26

**Authors:** Zhiqiang Han, Haijun Wang, Xin Liu, Zhige Tian, Qinglong Gong, Xiuli Zhang, Xiao Li, Rui Du, Xiaoliang Hu, Chao Xu

**Affiliations:** 1 College of Animal Science and Technology, College of Veterinary Medicine, Jilin Agricultural University, Changchun, China; 2 Jilin Provincial Academy of Forestry Sciences, Changchun, China; 3 Jilin Province Northeast Tiger Garden and Jilin Wild Animal Rescue Breeding Center Committee, Changchun, China; 4 Faculty of Agriculture, Forestry and Food Engineering, Yibin University, Yibin, China; 5 College of Veterinary Medicine, Jilin University, Changchun, China

**Keywords:** Amur tiger, canine coronavirus (CCoV), feline coronavirus, *Panthera tigris altaica*, recombination

## Abstract

Canine coronavirus (CCoV) is an important enteric alphacoronavirus primarily affecting canids. Here, we detected canine coronavirus RNA in a captive 9-year-old Amur tiger (*Panthera tigris altaica*) in China. The complete viral genome was obtained using metagenomic next-generation sequencing. Phylogenetic and recombination analyses were then performed to investigate its evolutionary relationship with canine and feline coronaviruses. The identified CCoV strain clustered within established canine coronavirus lineages and showed sequence evidence of recombination involving coronavirus strains previously reported in other carnivore species. Although the detection of viral RNA alone does not establish a causal relationship between CCoV infection and disease outcome, this study provides molecular evidence that Amur tigers are susceptible to canine coronavirus infection. These findings expand the known host range of CCoV and contribute to understanding the evolution and cross-species transmission potential of coronaviruses among carnivores.

## 1 Introduction

Coronaviruses are enveloped, positive-sense, single-stranded RNA viruses belonging to the family Coronaviridae. Coronaviruses are taxonomically classified into four genera: Alphacoronavirus, Betacoronavirus, Gammacoronavirus, and Deltacoronavirus. These viruses exhibit broad host tropism, with confirmed infections in humans, a wide range of mammalian species (including domestic and wild animals), and avian populations, highlighting their zoonotic potential and ecological adaptability (Lai, 1990). Among these genera, alphacoronaviruses are predominant in domestic and wild carnivores. In particular, feline coronavirus (FCoV), including its pathogenic form feline infectious peritonitis virus (FIPV), has been detected in both captive and wild felid populations, including tigers (*Panthera tigris*), lions (*Panthera leo*), and snow leopards (*Panthera uncia*), where it can cause enteric or systemic disease. Canine coronavirus (CCoV), another member of the genus Alphacoronavirus, mainly infects canids and is commonly associated with enteric diseases. It has been reported that recombination between CCoV and transmissible gastroenteritis virus (TGEV) of swine is associated with the emergence of pathogenic CCoV variants in dogs (Decaro et al., 2009). In addition, deletions or mutations in ORF3abc have been linked to the emergence of pantropic CCoV strains with systemic tropism (Tian et al., 2021; Chen et al., 2019; Decaro et al., 2007). Notably, betacoronaviruses such as SARS-CoV-2 have demonstrated the capacity for cross-species transmission, through sustained circulation. Given the increasing interfaces among wildlife, domestic animals, and humans, the risk of cross-species transmission of coronavirus has become a growing concern for wildlife conservation and public health.

## 2 The study

Here, we identified a coronavirus infection in a 9-year-old captive Amur tiger (*Panthera tigris altaica*) housed at a zoo in Jilin Province, China, which exhibited clinical signs including severe emaciation, diarrhea, and intestinal mucosal hemorrhage prior to death. The tiger’s captive environment and dietary conditions were examined, and no obvious environmental contamination or food spoilage was identified, suggesting that an infectious etiology could not be excluded.

Multiple tissue samples, including heart, liver, spleen, lung, kidney, intestine, and feces, were collected immediately following necropsy by veterinary staff and stored at −80 °C until further analysis. The DNA and RNA of these tissues were extracted, and real-time RT-PCR assays were performed to detect several common feline and canine pathogens, including feline calicivirus (FCV) (Symes et al., 2015), feline panleukopenia virus (FPV) (Zhang et al., 2024), FCoV (Safi et al., 2017), canine distemper virus (CDV) (Martins et al., 2024), feline leukemia virus (FeLV) (Martins et al., 2024), and CCoV. Among the screened pathogens, only CCoV was detected (Ct = 25), indicating the presence of viral RNA in the tested samples.

**Figure 1:**
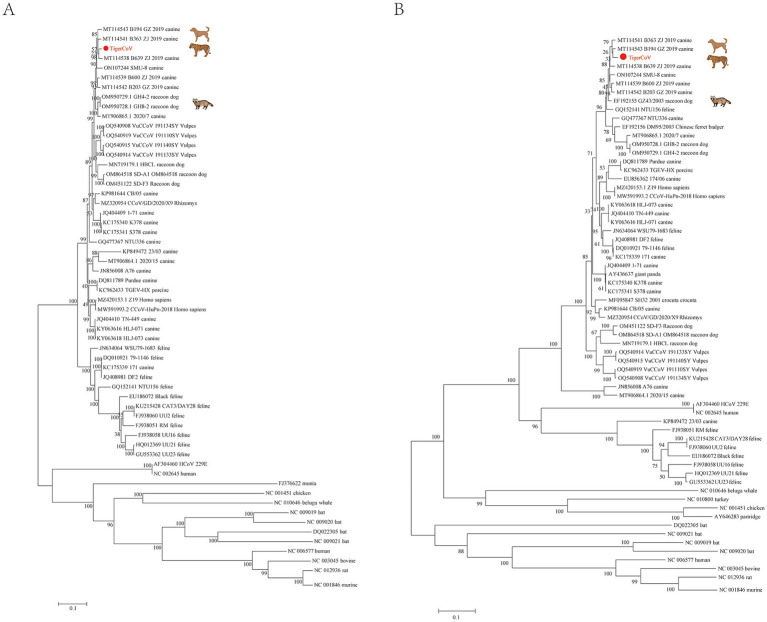
Phylogenetic analyses were performed on the canine coronavirus genome, and the S gene was detected in an Amur tiger. Sequencing generated a complete canine coronavirus genome, designated TigerCoV-JL1 and highlighted in red in the phylogenetic trees. **(A)** Phylogenetic tree was constructed using genome nucleotide sequences of the canine coronavirus (CCoV), transmissible gastroenteritis virus (TGEV), and feline coronavirus (FCoV). **(B)** Phylogenetic tree was generated based on the nucleotide sequences of the spike (S) gene of CCoV, TGEV, and FCoV.

**Figure 2:**
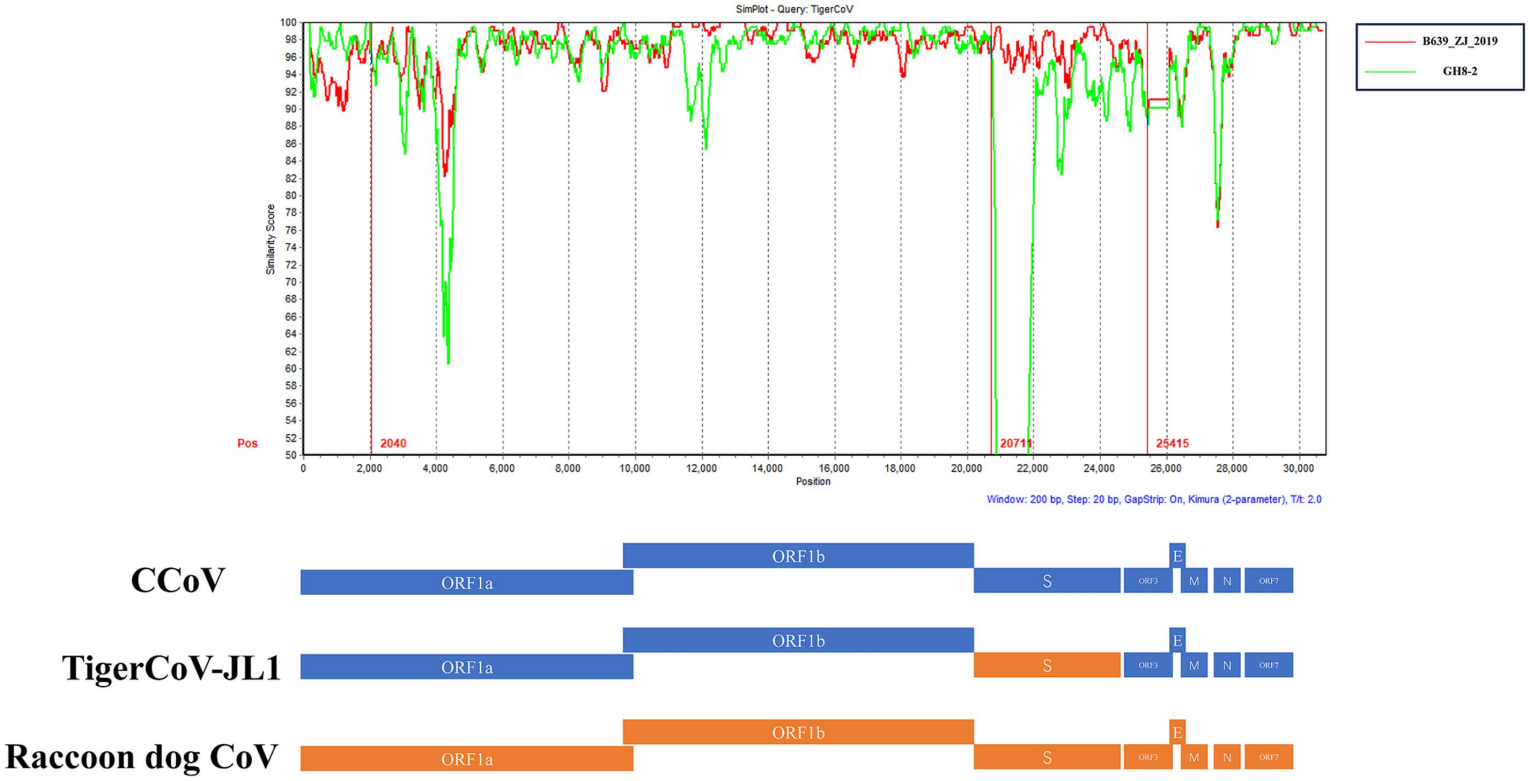
Similarity plot was generated based on the full-length genome sequence of TigerCoV-JL1. Complete sequences detected in the partial ORF1a and S gene regions of dog B639_ZJ_2019 and raccoon dog GH8_2 were used as reference sequences.

Furthermore, to obtain the complete genome sequence of the canine coronavirus TigerCoV-JL1, we performed metagenomic next-generation sequencing on extracted RNA for full-length genome sequencing as previously described (Liu et al., 2023). RNA libraries were prepared from the samples and sequenced on an Illumina MiniSeq platform using paired-end 150-cycle sequencing, as previously described (Liu et al., 2023). The raw sequencing data generated from this study were deposited in the Sequence Read Archive (SRA) under accession number PRJNA1232954. The complete genome sequence of TigerCoV-JL1 (Table 1) was submitted to GenBank (accession no. PV259885) and subsequently compared with previously reported canine and feline coronavirus strains. Phylogenetic analysis showed that TigerCoV-JL1 was clustered with domestic canine coronavirus (B639_ZJ_2019) (Figure 1A), while analysis based on the spike (S) gene indicated the closest genetic relatedness to the raccoon dog strain GZ43/2003 (Figure 1B). More importantly, the occurrence of recombination of TigerCoV-JL1 between B639_ZJ_2019 and the GH8_2 raccoon dog was detected at partial open reading frame 1a (ORF1a) and S genes, which revealed a novel canine-raccoon dog coronavirus (Figure 2).

**Table 1:** Primers used for completely sequencing the TigerCoV-JL1.

Name	Sequence (5′-3′)	Position
1-F	GTGAGTGTAGCGTGGCTATC	1–20
1-R	TCCTCAACAACCTTCCAG	1,500–1,517
2-F	TCTTAGTACTAACCTTTTTG	1,410–1,419
2-R	TTGATGTACCAGATTCAGAT	3,270–3,289
3-F	CTTTGATATCAAAAATCCAG	3,201–3,220
3-R	ACTTTTCTACTTTTGCGCAC	5,301–5,320
4-F	ATGGACAATGATTGTGAAAT	5,251–5,270
4-R	CTACAAAAGTTTTAACCAAG	7,341–7,360
5-F	CTCAACGTGGTAAAAGTGTT	7,271–7,290
5-R	GTTCTCGAAGTATGATTCGA	10,931–10,950
6-F	TGAGATTGTTCTAGAAAAGC	10,851–10,870
6-R	TCTGTTTTAACTATGTCATC	14,811–14,830
7-F	TGGACCTGACGGAGACTATT	14,741–14,760
7-R	ACTTCACTATCAAGATCAGT	18,741–18,760
8-F	TCGCAACATACAAGTTTGTG	18,661–18,680
8-R	ACAGTACACATCAGTATTAT	22,041–22,060
9-F	AGCTTCAACATTAAGTAACA	22,000–22,019
9-R	TCTTCTTCTTTTAAATCAAT	26,850–26,869
10-F	CTTCTCAATGGTGATTTTAT	25,760–25,779
10-R	GGGAGAGCGCGGCGACGGTC	29,317–29,336

## 3 Discussion

Previous studies have shown that multiple felid species, including the serval (*Leptailurus serval*), bobcat (*Lynx rufus*), puma (*Puma concolor*), cheetah (*Acinonyx jubatus*), and a tiger, can be infected with FCoV (Stout et al., 2021). In this study, a CCoV strain was identified in a deceased Amur tiger that exhibited clinical signs such as diarrhea and emaciation, which are also commonly observed in FCoV infections. These findings suggest that CCoV infection should be considered as a potential factor in the health assessment of Amur tigers. Phylogenetic and recombination analyses indicated that TigerCoV-JL1 clustered with domestic CCoV and exhibited recombination signals involving *S* gene regions related to coronaviruses previously detected in raccoon dogs. The results suggested that CCoV has undergone recombination with coronaviruses detected in felids and other carnivores and has experienced complex transmission pathways, highlighting the dynamic evolutionary processes shaping canine coronavirus. However, the identified recombination signals do not necessarily indicate that recombination occurred within the Amur tiger, and the virus may have originated in canine or other carnivore hosts prior to spillover (Kane et al., 2023; Gamble et al., 2023). Previous reports have indicated that CCoV can infect multiple host species, including foxes, raccoon dogs, and humans (Chen et al., 2019; Whittaker and Stout, 2022; Zahn et al., 2019). Such cross-species transmission may be facilitated by habitat overlap and foodborne exposure under captive or semi-captive conditions. In these settings, sympatric animals that are difficult to completely exclude, including feral cats, birds, and bats, may function as potential intermediate hosts through which indirect viral exposure to Amur tigers could occur. Our results provide molecular evidence that a canine coronavirus can infect an Amur tiger. It highlights the potential contribution of wild felids to cross-species transmission events involving canine-derived coronaviruses. Given the critical conservation status of Amur tigers, our findings underscore the need for comprehensive surveillance programs, improved biosecurity measures, and targeted conservation efforts to reduce the risk of emerging infectious diseases among wildlife populations.

Therefore, this study provides molecular evidence of infection with recombinant canine coronavirus in an endangered wild felid, which underscores the importance of interdisciplinary collaboration among conservation biologists, veterinarians, and public health experts. Further in-depth investigation and proactive management of cross-species pathogen transmission are essential for protecting biodiversity, maintaining ecosystem stability, and enhancing animal health globally.
